# Establishment of an Ex Vitro Rooting Strategy for *Monochasma savatieri* Increases Propagation Efficiency

**DOI:** 10.3390/plants15172638

**Published:** 2026-08-28

**Authors:** Suzhen Liu, Ting Zhang, Wei Huang, Shutong Sang, Mansheng Zeng, Jinping Zhang, Guisheng Luo, Yuling Zou, Fengqing Li, Xingliang Chen

**Affiliations:** 1 Experimental Center of Subtropical Forestry, Chinese Academy of Forestry, Fenyi 336600, China; lszylzx@163.com (S.L.); zhangting202607@126.com (T.Z.);; 2 Research Institute of Subtropical Forestry, Chinese Academy of Forestry, Hangzhou 311400, China

**Keywords:** *Monochasma savatieri*, micropropagation, plant growth regulators (PGRs), substrate physical properties, survival rate

## Abstract

*Monochasma savatieri* is an endangered hemiparasitic medicinal herb characterized by its broad host range. The ongoing decline of its natural resources highlights the urgent need for an efficient artificial propagation system. This study aimed to establish an optimized ex vitro rooting and acclimatization protocol through the assessment of plant growth regulators (PGRs), immersion durations, and substrate formulations. In vitro rooting on agar-solidified medium was suboptimal, thereby prompting the development of an ex vitro protocol. The major novelty of this work lies in the development of an integrated system that synergistically combines PGR pulse treatment with substrate physical property optimization, while completely eliminating the conventional in vitro agar-based rooting phase. Of the 18 PGR treatments screened, a 15 s immersion dip in NAA 480 mg·L^−1^ + IAA 720 mg·L^−1^ + GA 200 mg·L^−1^ (treatment 10) performed best, achieving a 95.45% survival rate four months after transplantation, a 92.96% overwintering survival rate, and the longest roots (125.49 mm). Quadratic regression analysis revealed that root length is the best predictor of survival, with an optimum of 94.7 mm. Additionally, substrate evaluation revealed that T12 (peat moss:perlite:sawdust:carbonized chaff = 3:1:3:3, *v*/*v*) achieved the highest survival rate (85.20%), which correlated with moderate aeration porosity (17.44%), high water-holding porosity (142.56%) and the highest total porosity (160.00%). The integrated protocol eliminates the in vitro rooting phase, thereby reducing both cost and labor while shortening the production cycle by at least one month. Collectively, this study establishes a complete and efficient ex vitro rooting acclimatization system for *M. savatieri*, providing a practical basis that may facilitate large-scale propagation and conservation of this threatened species.

## 1. Introduction

*Monochasma savatieri* Franch. ex Maxim. is a perennial herb belonging to the family Scrophulariaceae, with a distribution restricted to Southeast China and the Amakusa Islands of Kyushu, Japan [1]. The entire plant has been used in traditional Chinese medicine for the treatment of common cold, cough, bloody flux, menoxenia, toothache and mammary abscess [2]. The bioactive components of *M. savatieri* are primarily total phenylethanoid glycosides (TPG), which are present at high concentrations throughout the plant; to date, 11 phenylethanoid glycosides have been isolated. Recently, in vivo and in vitro studies have demonstrated that TPG has several biological properties, including antimicrobial, anti-inflammatory [3], antioxidant and myocardial ischemia injury protection effects [4]. However, the natural populations of *M. savatieri* in China have experienced a sharp decline, primarily driven by anthropogenic disturbances including habitat destruction and over-exploitation, as documented by our field surveys conducted from 2017 to 2020 [5]. Consistent with this trend, the species has also been categorized as endangered in Japan [6]. These conservation concerns underscore the urgent need for an efficient artificial propagation and cultivation system for this medicinal species [7].

Seed-based propagation of *M. savatieri* presents two major challenges. Although seeds of *M. savatieri* can be germinated in vitro under axenic conditions [7], conventional propagation via seed germination remains inefficient due to seed dormancy and the requirement for a host during early seedling establishment—a consequence of its root hemiparasitic nature [8]. Moreover, seed germination requires a specific microenvironment (sunny, moist, and shaded, similar to conditions preferred by certain mosses) and exhibits a sharply decreasing germination rate with prolonged storage. As an alternative, micropropagation of *M. savatieri* has been explored in several studies [7,9,10]. In the greenhouse, tissue-cultured *M. savatieri* plantlets were initially rooted in vermiculite (as a substitute for agar), then subsequently transferred to potting mix either with or without a host plant and acclimatized under high humidity conditions [10,11]. However, root induction in agar-solidified medium is notably difficult for *M. savatieri* and often results in the formation of non-functional aerial roots. This contributes to a high mortality rate during subsequent ex vitro transfer. Therefore, an integrated strategy combining ex vitro rooting with simultaneous acclimatization has emerged as a more reliable alternative to conventional micropropagation, offering the potential to bypass the agar-rooting phase entirely. This transition to ex vitro conditions remains among the most critical and challenging steps of the overall propagation protocol [12].

Two major factors influence the success of this ex vitro transition: substrate composition and plant growth regulator (PGR) application. Regarding the first factor, substrate composition plays a decisive role in determining both rooting efficiency and plantlet survival. The physical properties of the substrate—such as water-holding capacity, porosity and permeability—directly affect root system architecture [13]. Porous substrates, including vermiculite [14] and vermiculite–perlite mixtures [15], have been shown to promote rooting and enhance plantlet growth by increasing hydraulic conductivity and facilitating nutrient uptake from the culture medium [16]. *Sphagnum* moss and peat are the most commonly used substrates for light-substrate container seedling cultivation; however, environmental changes have reduced their availability, leading to declining plant quality and rising costs [17,18] and thus necessitating the search for alternative materials. To address this, current mixed substrates for light-substrate container seedlings increasingly incorporate agricultural and forestry wastes such as sawdust and chaff (which require composting to eliminate harmful substances). Sawdust and chaff are two major types of agricultural and forestry waste. Converting these wastes into organic substrates not only reduces reliance on *Sphagnum* moss and peat, but also alleviates environmental pressure, significantly lowers production costs and promotes sustainable economic and environmental development. In terms of functional properties, sawdust exhibits high water-holding capacity, whereas chaff offers high aeration capacity but low water sorption capacity. However, no single substrate material is optimal in all situations, owing to variability in physicochemical properties and the specific requirements of different plant varieties or cultivars [15].

Beyond substrate properties, the application of appropriate plant growth regulators (PGRs)—particularly auxins—constitutes another critical factor determining transplanting success. Auxins are essential for inducing root primordium initiation and promoting subsequent root elongation, thereby ensuring the production of high-quality plantlets with well-developed root systems. The synergistic interaction between substrate conditions and exogenous auxin application ultimately determines the overall efficiency of the ex vitro rooting and acclimatization process [19]. Due to the activity of auxins, such as indole-3-acetic acid (IAA), 1-naphthaleneacetic acid (NAA), and indole-3-butyric acid (IBA), single-auxin treatments or their combinations have proven to enhance root induction. NAA has been reported to positively affect root number, while IBA has been confirmed to promote root elongation in several studies, including those on *Momordica dioica* and *Hildegardia populifolia* [20,21]. Beyond auxins, gibberellins (GAs) also play a role in adventitious root development, although their effects are more context-dependent. Studies have shown that GAs generally inhibit adventitious root initiation but can stimulate root elongation once primordia are established [7,8]. In tobacco, proper GA localization in vascular tissue was found to be required for the emergence and subsequent elongation of adventitious roots [7], while in hybrid aspen and *Arabidopsis*, GA treatment negatively affected the number of adventitious roots produced, primarily by perturbing polar auxin transport [8]. In *M. savatieri*, combined application of cytokinins and auxins has also demonstrated a positive effect on ex vitro root induction [11].

Despite these advances, systematic optimization of the combined effects of auxin concentration, soaking duration, and substrate type on ex vitro rooting and acclimatization in *M. savatieri* has not yet been reported. Furthermore, the integration of rooting and acclimatization into a single step—aimed at reducing time, labor and cost—remains unexplored for this species.

In the present study, we aimed to develop an optimized ex vitro rooting and acclimatization system for *M. savatieri* by integrating PGR treatment and substrate selection into a streamlined cost- and labor-efficient protocol. Specifically, we assessed the impact of the substrate on rooting performance of *M. savatieri* and optimized the conditions for *ex vitro* rooting and simultaneous acclimatization—including the concentrations of different auxins, soaking duration and substrate composition to develop a practical protocol suitable for large-scale application. This integrated approach simplifies the conventional multi-step process and ensures the production of robust, well-rooted plantlets suitable for field cultivation. 

## 2. Results

### 2.1. In Vitro Rooting and Acclimatization

The in vitro rooting performance of *M. savatieri* varied markedly depending on auxin type and concentration (Table 1). Among all treatments, 1.0 mg·L^−1^ ABT (20% NAA + 30% IAA as active ingredients; Beijing ai-bidi Biotechnology Co. Ltd, Beijing, China) achieved the highest rooting frequency (61.66%), while rooting frequencies on other media ranged from 30.51% to 42.48%, all significantly lower. For root length, IAA generally outperformed ABT and NAA: the longest roots (5.21 cm) were obtained with 1.0 mg·L^−1^ IAA, whereas ABT-supplemented media produced roots ranging from 0.76 cm (at 0.8 mg·L^−1^) to 2.07 cm (at 0.2 mg·L^−1^). However, IAA-treated plantlets exhibited substantially lower rooting frequencies (38.24% at 1.0 mg·L^−1^) and greater variability, whereas ABT provided a more balanced combination of acceptable rooting frequency and adequate root length for transplanting. Plant height was also affected by the auxin type and concentration. The tallest plants were obtained by supplementing the medium with 1.0 mg·L^−1^ IAA (6.09 cm). Significantly shorter plants were produced on media supplemented with 0.2–0.8 mg·L^−1^ IAA and 0.8–1.0 mg·L^−1^ ABT. Notably, shoots cultured on hormone-free medium (0 mg·L^−1^) produced only thin, non-functional aerial roots that failed to establish a functional root–soil interface, resulting in >80% mortality within two weeks after transfer to substrate. 

Based on the comprehensive evaluation of rooting frequency, root length, and plant height, 1.0 mg·L^−1^ ABT was selected as the optimal auxin treatment for subsequent ex vitro optimization, as it offered the highest rooting success and adequate root quality despite not producing the longest roots. However, the specific concentrations of NAA and IAA, as well as the optimal immersion duration for ex vitro application, remained to be determined. Therefore, we next shifted our focus to an ex vitro rooting strategy to optimize these parameters.

To evaluate the interactive effects of auxin type and concentration on in vitro rooting performance, a two-way ANOVA was performed. The results revealed that auxin type significantly affected root number, root length, and plant height (*p* < 0.05), while concentration significantly influenced root length (*p* < 0.05). More importantly, the interaction between auxin type and concentration had significant effects on rooting frequency and plant height (*p* < 0.05). These findings indicate that the optimal auxin treatment cannot be determined by either factor alone, confirming the need for empirical screening of auxin type–concentration combinations (Table S1).

### 2.2. Optimization of Ex Vitro Rooting and Acclimatization

#### 2.2.1. Effect of PGR Concentration and Immersion Time on Seedling Growth

**Figure 1:**
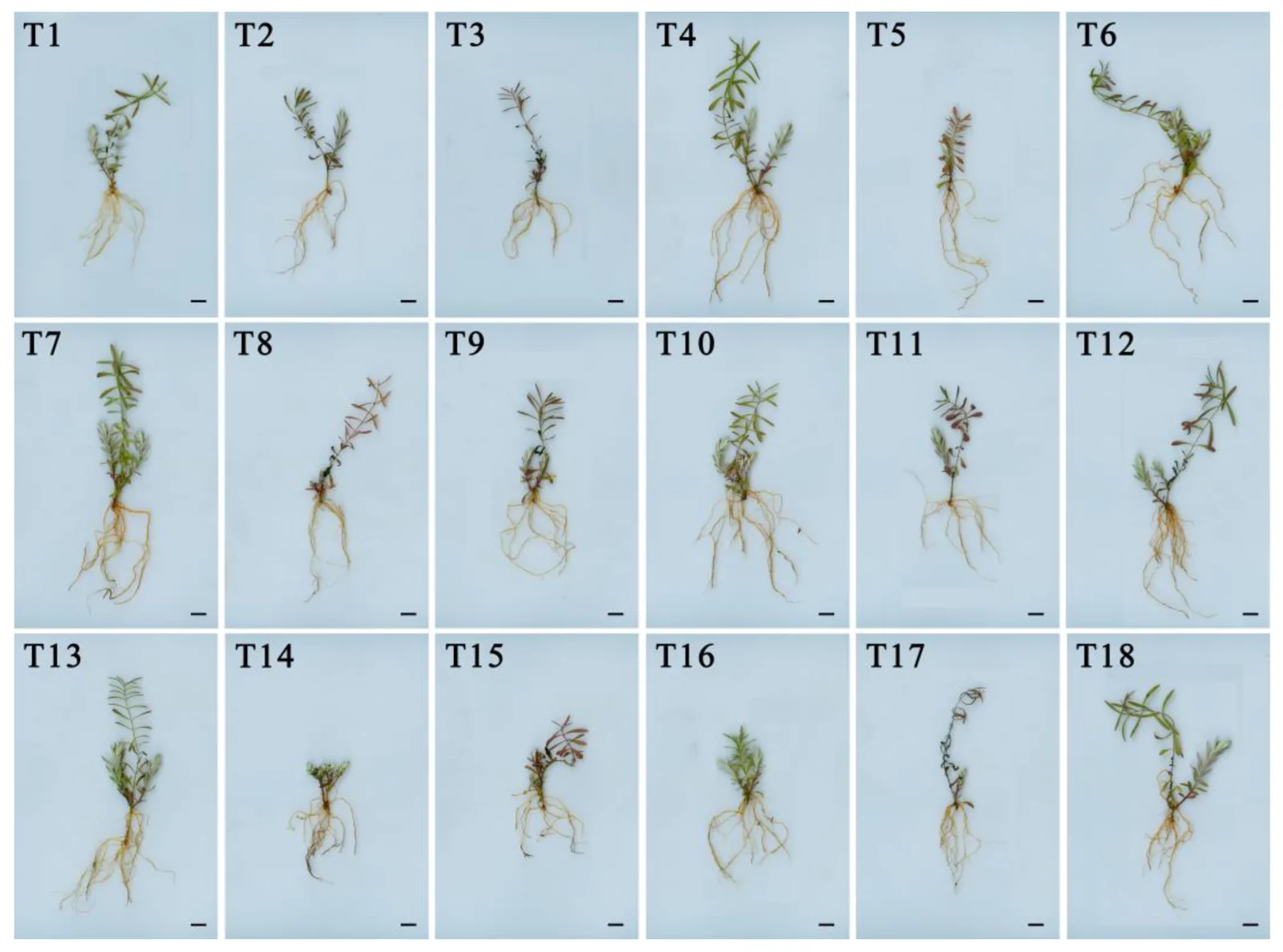
Representative images of *M. savatieri* plantlets at 6 months after ex vitro rooting. T1–T18 correspond to the treatments with NAA/IAA/GA_3_ concentrations (mg·L^−1^) and immersion times (s) as follows: T1 = 120/180/50, 15 s; T2 = 120/180/50, 30 s; T3 = 120/180/50, 60 s; T4 = 240/360/100, 15 s; T5 = 240/360/100, 30 s; T6 = 240/360/100, 60 s; T7 = 360/540/150, 15 s; T8 = 360/540/150, 30 s; T9 = 360/540/150, 60 s; T10 = 480/720/200, 15 s; T11 = 480/720/200, 30 s; T12 = 480/720/200, 60 s; T13 = 600/900/250, 15 s; T14 = 600/900/250, 30 s; T15 = 600/900/250, 60 s; T16 = 720/1080/300, 15 s; T17 = 720/1080/300, 30 s; T18 = 720/1080/300, 60 s. Scale bars = 1 cm.

**Figure 2:**
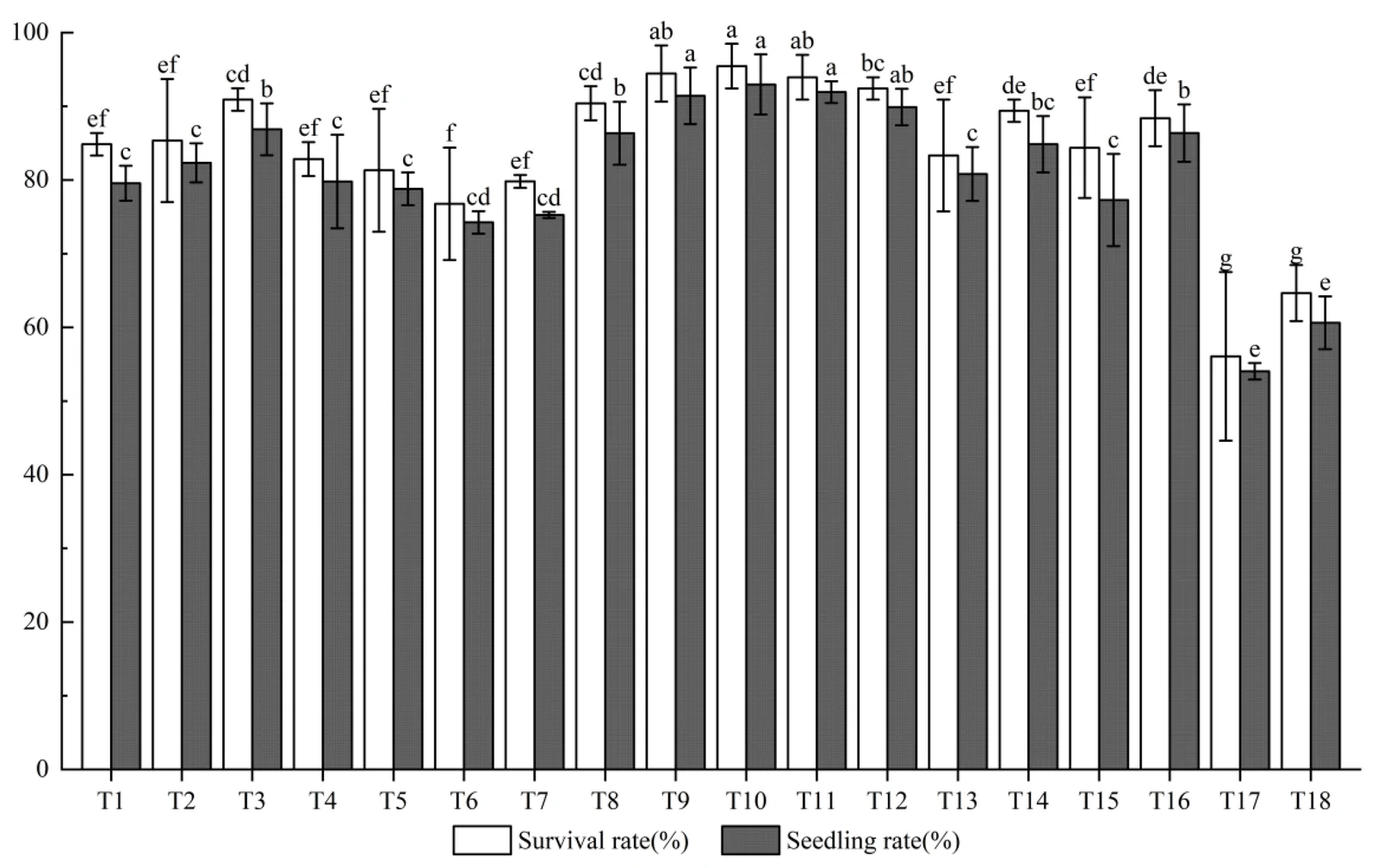
Four-month survival and next-year seedling rates of *M. savatieri* across 18 treatments detailed in Table 2. Error bars represent standard deviation (SD). Different lowercase letters above bars indicate significant differences among treatments at *p* < 0.05 (Duncan’s multiple range test).

The effects of varying concentrations of NAA, IAA and GA, in combination with three immersion durations, on ex vitro rooting and subsequent survival of *M. savatieri* are summarized in Table 2, Figure 1 and Figure 2.

Among the 18 treatment combinations, treatment 10 (NAA 480 mg·L^−1^ + IAA 720 mg·L^−1^ + GA 200 mg·L^−1^, 15 s immersion) yielded the highest four-month survival rate (95.45%) and the highest next-year seedling rate (92.96%) (defined as the percentage of plantlets that successfully overwintered and resumed growth, see Section 4.2.2). This survival rate was significantly higher than that obtained with any other treatment (*p* < 0.05). Treatment 10 also produced the greatest root length (125.49 mm), significantly longer than those recorded in treatments 1–9 and 11–18 (*p* < 0.05), accompanied by a relatively low number of root tips (101.17) and the smallest mean root diameter (0.23 cm).

In contrast, the highest tested PGR concentrations (treatments 17 and 18: NAA 720 mg·L^−1^ + IAA 1080 mg·L^−1^ + GA 300 mg·L^−1^) resulted in markedly lower four-month survival rates of 56.06% and 64.65%, respectively, both significantly lower than that of treatment 10 (*p* < 0.01). At the lowest PGR levels (treatments 1–3), survival rates ranged from 65.15% to 69.70%, with no significant differences among treatments 1, 2 and 3 (*p* > 0.05). Among these, treatment 3 exhibited the highest number of root tips (307.50) but the shortest root length (49.61 mm); this root number was significantly higher than that of treatment 10 (*p* < 0.05), whereas root length was significantly shorter (*p* < 0.05).

Further analysis of treatments sharing the same PGR composition (treatments 10, 11 and 12, with immersion times of 15, 30 and 60 s, respectively) revealed a progressive change in root morphology with increasing soaking duration. As immersion time increased, four-month survival decreased slightly from 95.45% to 92.42%, but the differences among the three treatments were not statistically significant (*p* > 0.05). Meanwhile, mean root diameter and volume increased progressively. Treatment 12 recorded the largest root diameter (0.41 cm) and the greatest root volume (0.17 cm^3^) among all treatments, both significantly larger than those of treatment 10 (*p* < 0.05).

Additionally, treatment 15 (NAA 600 mg·L^−1^ + IAA 900 mg·L^−1^ + GA 250 mg·L^−1^, 60 s) produced the second highest root number (189.83) but the shortest root length (40.54 mm) across all treatments, with a survival rate of 84.38% that was not significantly different from treatments 1–3 (*p* > 0.05). Overall, the data demonstrate that moderate PGR concentrations combined with a brief immersion period (treatment 10) maximized both survival and root elongation. Conversely, excessively high PGR concentrations or prolonged immersion reduced survival, and root number alone was not a reliable predictor of successful establishment.

To further examine the interactive effects of PGR concentration and immersion duration on plantlet survival, a two-way ANOVA was performed using data from clone ZJLS11. PGR concentration significantly affected survival rate, seedling rate, bud number, and plant height (*p* < 0.05), whereas immersion time only significantly affected plant height (*p* < 0.05). The interaction between concentration and immersion time had highly significant effects on survival rate and seedling rate (*p* < 0.01), as well as on plant height (*p* < 0.05). These results confirm that PGR concentration is the dominant factor affecting ex vitro survival, and that immersion time modulates its effects primarily through interaction with concentration (Table S2).

#### 2.2.2. Physical Properties of Different Combined Substrates

**Figure 3:**
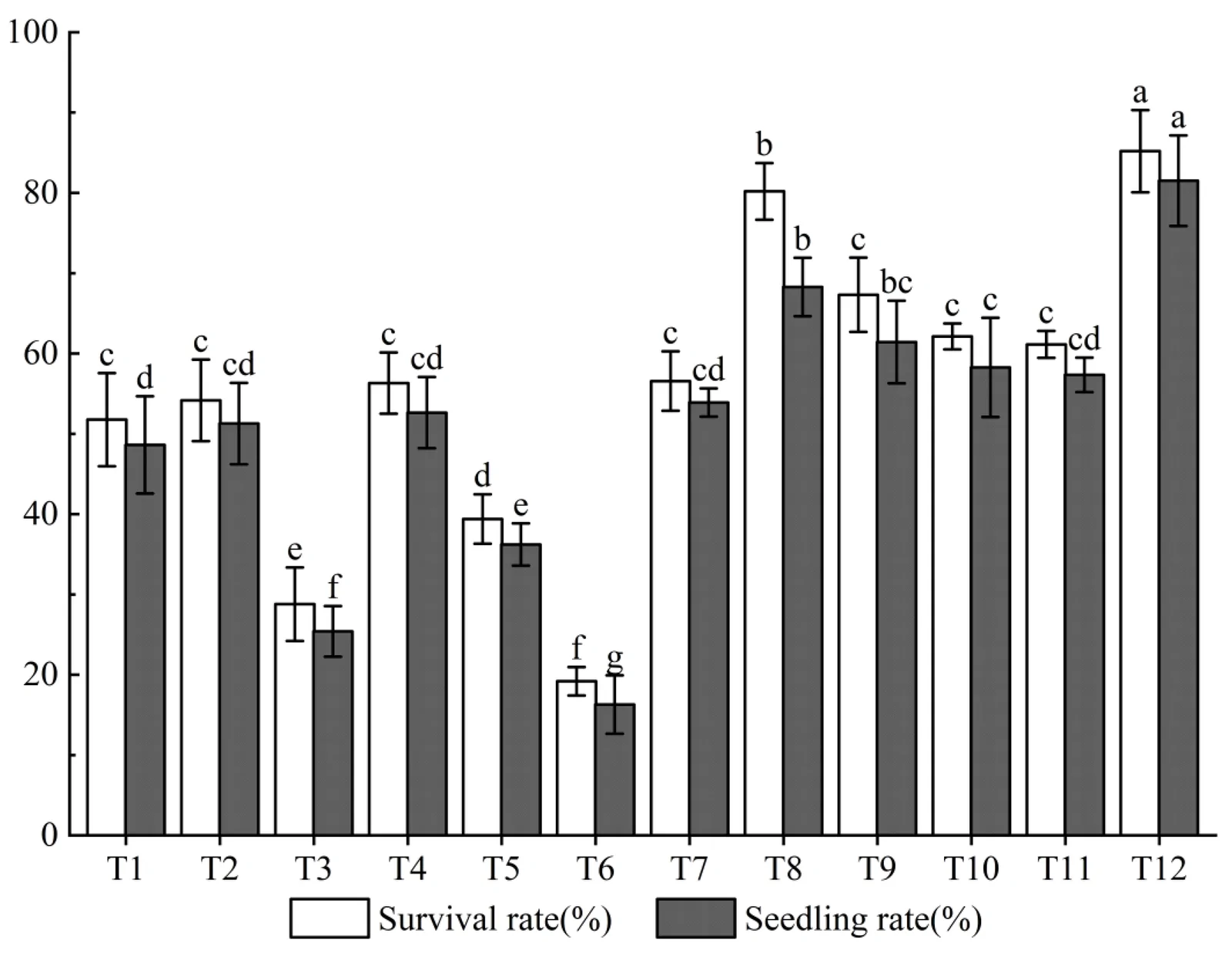
Four-month survival rates and next-year seedling rates of *M. savatieri* across twelve substrates. Substrate compositions and physical properties for T1–T12 are detailed in Table 3. The ‘next-year seedling rate’ was recorded in the following spring after overwintering and represents the percentage of the originally transplanted vegetative plantlets (R0 generation) that survived and resumed growth; this parameter does not refer to seed-derived R1 progeny. Error bars represent standard deviation (SD). Different lowercase letters above bars indicate significant differences among substrates at *p* < 0.05 (Duncan’s multiple range test).

Having identified the optimal PGR treatment (treatment 10), we next evaluated the effect of substrate composition on plantlet survival. The physical properties of twelve substrate formulations are presented in Table 3, and their corresponding effects on four-month survival and next-year seedling rates of *M. savatieri* are shown in Figure 3. Clear differences in plant performance were observed across substrates, which were closely associated with variations in substrate physical characteristics.

Substrate T12 achieved the highest four-month survival rate (85.20%) and next-year seedling rate (81.52%), both significantly higher than those of all other substrates (*p* < 0.05). Its physical properties are as follows: bulk density 0.45 g·cm^−3^, the highest total porosity (160.00%), aeration porosity 17.44%, water-holding porosity 142.56%, and a void ratio of 0.122. Substrate T8 ranked second, with a survival rate of 80.19% and a seedling rate of 68.28%. Its survival rate was significantly lower than that of T12 (*p* < 0.05) but significantly higher than those of the remaining substrates (*p* < 0.05). T8 has a higher bulk density (0.58 g·cm^−3^), total porosity of 136.00%, but very low aeration porosity (5.74%)—among the lowest of all substrates—and water-holding porosity of 130.26%.

Intermediate-performing substrates included T4 (survival 56.31%), T7 (56.57%), T2 (54.17%) and T11 (61.13%). No significant differences were detected among these four (*p* > 0.05), but all were significantly lower than T8 and T12 (*p* < 0.05). Among them, T7 exhibits the highest water-holding capacity (563.43%) and the lowest aeration porosity (5.70%), while T11 has the highest aeration porosity (44.54%) and the lowest bulk density (0.31 g·cm^−3^). Despite these contrasting properties, neither T7 nor T11 exceeded 62% survival.

The poorest performing substrates were T6 (survival 19.19%, seedling 16.29%) and T3 (survival 28.79%), both significantly lower than all other substrates (*p* < 0.01). T6 has a total porosity of 149.50%, relatively high aeration porosity (38.77%), but water-holding porosity of only 110.73%. T3 shows a bulk density of 0.56 g·cm^−3^, aeration porosity of 22.75%, and water-holding porosity of 117.76%.

Notably, neither extremely low aeration porosity (e.g., T4, T7, T8, T10 all below 10%) nor insufficient water-holding porosity (e.g., T1, T2, T3) resulted in the highest survival. T12, with only moderate aeration porosity (17.44%) but high water-holding porosity (142.56%) and the highest total porosity (160.00%), significantly outperformed substrates with better aeration (e.g., T11) or higher water retention (e.g., T7).

In summary, T12 was identified as the most suitable substrate for ex vitro rooting and acclimatization of *M. savatieri*, whereas T6 and T3 were the least effective.

### 2.3. Quadratic Regression Analysis of Root Traits in Relation to Survival Rate

To further understand how root morphology influences transplant success, quadratic polynomial regressions (forced through the origin) were performed with survival rate as the dependent variable and root length, root area and root diameter as independent variables. The decision to force the regression through the origin was based on the biological assumption that zero root length, area or diameter would result in zero survival. However, we acknowledge that this assumption may be overly strict, and readers should interpret the optimized values with this limitation in mind. The results are summarized in Table 4.

Root length showed a significant inverted U-shaped relationship with survival rate (*R*^2^ *= 0.977*, *F = 344.64*, *p < 0.001*), with the following regression equation:

Survival rate = 2.083 × Root length − 0.011 × (Root length)^2^ (1)

According to this equation, the predicted survival rate reached its maximum (approximately 98.6%) when root length was about 94.7 mm. Both lower and higher root lengths reduced the predicted survival rate, indicating an optimal root length range.

Root area also exhibited a significant quadratic relationship (*R*^2^ *= 0.976*, *F = 334.43*, *p < 0.001*):

Survival rate = 22.671 × Root area − 1.390 × (Root area)^2^ (2)

The optimum root area was about 8.15 cm^2^, corresponding to a predicted survival rate of approximately 92.4%. Roots having a surface area that is either too small or too large is detrimental to survival.

Root diameter likewise yielded a significant quadratic model (*R*^2^ *= 0.984*, *F = 497.09*, *p < 0.001*):

Survival rate = 495.727 × Root diameter − 707.777 × (Root diameter)^2^ (3)

The optimal root diameter was about 0.35 cm, with a predicted survival rate of about 86.7%. The absolute value of the quadratic coefficient for the root diameter was very large, indicating that survival rate was highly sensitive to changes in root diameter, and any deviation from the optimum would cause a sharp decline in survival.

Overall, root length, area and diameter all exhibited significant non-linear relationships with survival rate. Among them, root length gave the highest goodness-of-fit (*R*^2^ *= 0.977*) and its predicted maximum did not exceed the biological limit of 100%, making root length the best indicator for predicting survival rate. In line with these results, Treatment 10 (NAA 480 mg·L^−1^ + IAA 720 mg·L^−1^ + GA 200 mg·L^−1^, 15 s immersing) resulted in a root length of 125.49 mm, slightly above the optimum, yet its survival rate remained as high as 95.45%; in contrast, Treatment 3 (low PGR concentration, 60 s dipping) resulted in a root length of only 49.61 mm and showed a significantly lower survival rate (Figure 2). These observations further validate the inverted U-shaped relationship between root length and survival rate.

## 3. Discussion

### 3.1. Limitations of In Vitro Rooting and Advantages of Ex Vitro Rooting in M. savatieri

Conventional micropropagation of *M. savatieri* is severely limited by the poor in vitro rooting performance of this species. This is evidenced by the fact that shoots cultured without any PGRs produce only morphologically aberrant aerial roots and fail to develop transplantable root systems; these preliminary observations justified the omission of a 0 mg·L^−1^ PGR control in our formal factorial screening, as the primary goal was to identify auxin treatments that generate functional, soil-adapted roots. However, even with auxin supplementation, in vitro rooting remained suboptimal. Although 1.0 mg·L^−1^ ABT gave the highest rooting frequency (61.66%) on agar-solidified medium (Table 1), most induced roots were aerial and morphologically fragile. This phenomenon is not uncommon in hemiparasitic plants such as *M. savatieri*, which depend on host plants for water and nutrient acquisition under natural conditions. Similar rooting difficulties have been reported in other hemiparasitic plants. For instance, *Castilleja tenuiflora*, another hemiparasitic member of the Orobanchaceae family, develops small and thin roots during micropropagation, significantly hindering the process [22]. Likewise, in *Orthocarpus species*, stem tips cultured in vitro exhibit poor root development in the absence of host-derived factors [23]. The high humidity and low mechanical resistance of agar-gelled media fail to promote the differentiation of roots that are adapted to soil conditions.

In sharp contrast, the optimized ex vitro rooting protocol (treatment 10: NAA 480 + IAA720 + GA200 mg·L^−1^, 15 s immersion) yielded a four-month survival rate of 95.45% and a next-year seedling rate of 92.96% (Figure 2), with roots reaching mean lengths of 125.49 mm and a mean diameter of 0.23 cm (Table 2). The ex vitro system combines rooting and acclimatization into a single step by directly planting shoots into a porous substrate, thus omitting the agar phase and avoiding the stress associated with washing and transplanting. 

The cost, time and labor savings of ex vitro systems compared to their in vitro counterparts have been well documented in other species, and the present findings extend these benefits to *M. savatieri* [24]. For example, ex vitro rooted plantlets with concurrent acclimatization were successfully achieved in *Moringa concanensis* by pre-treating shoot bases with IBA at 250 ppm for 5 min [25]. In *Hildegardia populifolia*, a critically endangered medicinal tree, ex vitro rooting with simultaneous acclimatization resulted in a 98.33% survival rate in Soilrite [21]. For *Bauhinia racemosa*, ex vitro rooting with 400 mg·L^−1^ IBA for 7 min on sterile Soilrite achieved approximately 85% survival after greenhouse hardening [26]. The superior performance of ex vitro systems is largely attributable to the fact that roots formed in porous substrates are more branched and physiologically functional than those induced on agar-solidified media. 

In addition to increased performance, there are also cost-cutting benefits to ex vitro systems. First, ex vitro rooting eliminates costly agar, sucrose and sterile room maintenance, requiring only reusable PGR solutions and low-cost substrate. Second, it shortens the production cycle by at least one month. Third, it reduces labor by omitting agar washing and individual potting; shoots are simply dipped in PGR and inserted into substrate bags, facilitating scaling.

To our knowledge, this study represents the first systematic optimization of ex vitro rooting for *M. savatieri* that simultaneously integrates both PGR treatment and substrate selection. Previous studies either relied on in vitro rooting using vermiculite as a supporting material or required co-culture with a host plant to achieve acceptable survival rates [7,10]. This approach is particularly important for endangered medicinal plants such as *Nardostachys jatamansi*, where efficient micropropagation combined with ex vitro acclimatization is essential for ex situ conservation and reintroduction efforts [27]. This strategy effectively bypasses the formation of aerial roots and provides a scalable solution for the large-scale propagation of this endangered species. Having established the superiority of the ex vitro approach, we next examine the specific effects of PGR concentration and immersion duration on root development.

### 3.2. Effects of PGR Type, Concentration and Immersion Duration on Root Architecture and Survival Rate

The optimization of PGRs is critical for successful ex vitro rooting. In this study, we observed a clear non-linear relationship among PGR concentration, immersion time, root morphology and post-transplant survival. During the in vitro phase (Table 1), ABT at 1.0 mg·L^−1^ produced the highest rooting frequency (61.66%) but resulted in short roots (1.68 cm); IAA at the same concentration yielded longer roots (5.21 cm) but a lower rooting percentage (38.24%); NAA consistently induced only short roots. These results suggested that ABT is more effective in promoting root initiation, while IAA preferentially stimulates root elongation. ABT is widely recognized as a dual-function auxin preparation that not only supplies exogenous auxins but also enhances the synthesis of endogenous auxins in plant tissues [28]. This dual mechanism likely explains its superior performance in promoting root initiation in *M. savatieri* during the in vitro phase. For example, Guo et al. reported that NAA increased root number but suppressed elongation in *Polygonatum cyrtonema*, whereas IBA (an IAA analog) enhanced root length in *Momordica dioica* [19,29]. The poor performance of NAA in promoting root elongation may be explained by its higher chemical stability and stronger auxin activity, both of which can induce ethylene biosynthesis at elevated concentrations, thereby inhibiting cell expansion. Prolonged exposure to higher concentrations of IAA or IBA may lead to thinner, less robust roots, whereas moderate concentrations stimulate balanced root development [30,31].

In our ex vitro experiments, the highest PGR concentrations (T17 and T18) drastically reduced survival rates to 56–65% (Figure 2), strongly indicating phytotoxic effects. High levels of exogenous auxins have been reported to disrupt endogenous hormone homeostasis, induce aberrant cell division, and stimulate ethylene production, which is known to inhibit root elongation and promote premature senescence [32,33]. In *Oldenlandia corymbosa*, the optimal rooting response was achieved with 2.0 mg·L^−1^ IBA in combination with activated charcoal, whereas higher concentrations proved detrimental [34]. In *Rosa chinensis* cuttings, the rooting process is tightly regulated by the balance between IAA, ABA, GA_3_ and zeatin riboside (ZR) levels, with successful adventitious root formation requiring a relatively high IAA/ABA ratio during the early induction phase [35]. The same study reported that NAA and ABT treatments altered the levels and ratios of these endogenous hormones in cutting tissues, demonstrating that exogenous auxins indirectly regulate rooting by modulating the internal hormonal balance [35]. Similar supra-optimal inhibition effects have been reported in *Cadaba fruticosa* [36]. Furthermore, in *Spigelia marilandica* L., ex vitro rooting significantly reduced losses during acclimatization compared with in vitro rooting, highlighting the importance of optimizing PGR application strategies [24].

Treatments 10, 11 and 12 shared identical PGR concentrations (NAA 480 + IAA 720 + GA200 mg·L^−1^) but differed in immersion time (15, 30 and 60 s). As immersion time increased, root diameter and volume progressively increased from 0.23 cm and 0.07 cm^3^ (treatment 10) to 0.41 cm and 0.17 cm^3^ (treatment 12) (Table 2). Meanwhile, four-month survival rate declined slightly from 95.45% to 92.42%, although the difference was not statistically significant. The 15 s immersion produced the longest roots (125.49 mm) with the smallest diameter, whereas the 60 s immersion resulted in shorter and thicker roots. This suggests that a brief pulse of PGRs preferentially stimulates longitudinal root growth, whereas prolonged exposure shifts development toward radial thickening—a pattern that may be attributable, at least in part, to the over-accumulation of auxins and the consequent activation of genes involved in transverse cell expansion, although this hypothesis was not directly tested in the present study. Thicker roots generally exhibit lower specific root length and reduced surface area per unit dry mass, which might limit water and nutrient uptake efficiency. Although the survival rate difference between treatments 10 and 12 was not statistically significant, the observed trend of slightly lower survival in plants with thicker roots (92.42% vs. 95.45%) is consistent with this interpretation. 

Although auxins are primarily responsible for root initiation, the inclusion of GA_3_ in treatment 10 may have contributed to the observed root elongation. Gibberellins play a stage-dependent role in adventitious rooting: they can inhibit primordium initiation but promote the subsequent elongation of emerged roots [37,38]. Thus, the brief 15 s GA_3_ pulse likely acted primarily during the elongation phase, complementing auxin-driven initiation. The resulting long roots (125.49 mm) would facilitate substrate exploration and water uptake during acclimatization, supporting the high survival rate observed. The inclusion of GA probably promotes elongation [39], as GA_3_ content has been positively correlated with callus induction and adventitious root formation in *Rhododendron stamineum* [40].

Treatment 3 (lowest concentration, 60 s) produced the highest root tip number (307.50) but the shortest root length (49.61 mm) and yielded a survival rate of only 69.70% (Figure 2). This pattern confirms that root tip number, by itself, is a poor predictor of successful establishment. Instead, the functional transition and structural integrity of the root system are more critical for post-transplant survival [12,41]. As highlighted by Hiti Bandaralage et al., even after roots are initiated in vitro, plant survival during acclimatization remains very poor because of the few, mostly unbranched roots produced at the rooting stage [42]. Subsequent histological investigations revealed that acclimation success is positively correlated with the presence of fully differentiated secondary xylem in the root [43]. Our quadratic regression analysis (Table 4) identified root length as the best survival predictor (optimum 94.7 mm, *R*^2^ = 0.977). Short roots cannot effectively explore the substrate or take up water during the high-demand acclimatization period.

In summary, moderate PGR concentrations combined with a short immersion time (T10) achieve the optimal balance between root elongation, diameter and survival rate. Excessive concentrations induce toxicity or undesirable thickening, while insufficient levels produce numerous but non-functional short roots. With the PGR conditions optimized, we next consider how substrate physical properties further modulate rooting success.

### 3.3. Integrated Analysis: Substrate Physical Properties, Root Traits, and Their Non-Linear Effects on Survival

The physical environment of the rooting substrate directly shapes the root system architecture, which in turn determines the post-transplant survival of *M. savatieri*. Our results demonstrate that neither substrate properties nor root traits act linearly; optimal survival is achieved only when both factors fall within specific ranges, as demonstrated by the quadratic regression models (Table 4) and substrate performance (Table 3, Figure 3).

Substrate T12 (peat:perlite:sawdust:carbonized chaff = 3:1:3:3) exhibited the most balanced physical properties: a bulk density 0.45 g·cm^−3^, the highest total porosity (160.0%), moderate aeration porosity (17.44%), and high water-holding porosity (142.56%). This balanced formulation yielded the highest four-month survival (85.20%) and next-year seedling rate (81.52%) (Figure 3). Blok and Wever emphasized that bulk density, total pore space, and structural stability influence plant growth primarily through their effects on resistance to rooting, water retention and gas transport [44]. In contrast, substrates with extreme properties performed poorly: T7 (extremely low aeration, 5.70%) caused root hypoxia (survival 56.57%); T11 (excessively high aeration, 44.54%; low water-holding) induced moisture stress (survival 61.13%); and T6 and T3 gave the lowest survival (19.19% and 28.79%) due to insufficient water retention. These results confirm that *M. savatieri* requires high water-holding capacity with moderate aeration, which is consistent with its natural habitat in moist, shaded environments.

Quadratic regression analysis (Table 4) revealed significant inverted U-shaped relationships between root traits and survival rates. Root length was the best predictor (*R*^2^ = 0.977), with an optimum of 94.7 mm (predicted survival of 98.6%). Excessively long roots (>125 mm) may entail higher maintenance costs, whereas overly short or overly thick roots compromise both water and nutrient uptake. The superior performance of T12 is attributed to its ability to promote root elongation toward the optimum—mean root length in treatment 10 reached 125.49 mm, slightly above the optimum but still within the high-survival region. Poor survival in T3/T6 (root lengths only 49.61 and 40.54 mm) exactly matches model predictions. McClelland et al. provided key insights into why root quality matters beyond simple quantitative measures: at least 50% of post-transplant in vitro-produced adventitious roots either died immediately or persisted without further growth, and surviving roots required cortical collapse and new extensions before they could develop their secondary vascular tissues [41]. Economou et al. further noted that rooting in vitro in inert substrates with air-filled porosity is more advantageous than using agar-solidified medium, and that roots formed by dipping microcuttings into auxins are usually more branched than those produced from auxin supplementation in the culture medium [44]. In our study, the ex vitro system inherently selected for roots with greater functional integrity, as confirmed by the high survival rates of T12-grown plantlets.

Thus, integrating substrate physical analysis with quadratic regression offers a predictive framework for optimizing ex vitro propagation. Root length is a reliable and practical indicator for predicting ex vitro survival rate, and it is superior to the number or diameter of roots. This approach may be extendable beyond *M. savatieri* to other endangered species by combining root trait optimization with substrate selection, although its applicability would need to be validated on a case-by-case basis. In conclusion, substrate properties shape root traits, and root traits determine survival through well-defined non-linear functions—providing a practical tool for high-throughput screening of substrates or PGR protocols.

### 3.4. Integrated Optimal Protocol and Its Implications for Production and Conservation

By integrating the results of the two sequential experiments, a complete ex vitro rooting and acclimatization protocol for *M. savatieri* was established. The optimal treatment was treatment 10 (15 s immersion in NAA 480 mg·L^−1^ + IAA 720 mg·L^−1^ + GA 200 mg·L^−1^) combined with substrate T12 (peat:perlite:sawdust:carbonized chaff = 3:1:3:3). This yielded a four-month survival of 95.45% and a next-year seedling rate of 92.96% (Figure 2), and with T12 the survival reached 85.20% (Figure 3). Thus, the integrated system reliably achieves >85% establishment with high root quality (root length ~125 mm, diameter ~0.23 cm).

Compared with previous reports, our protocol offers several distinct advantages. Li et al. [11] used a combination of benzyladenine (BA) and auxins for ex vitro rooting, whereas we show that a brief auxin pulse alone is sufficient, thereby simplifying PGR use and reducing potential toxicity. Unlike the in vitro rooting methods of Yang [9] and Zhang et al. [10], our system eliminates the agar-based rooting phase, thereby avoiding aerial roots and post-transfer mortality. From a practical perspective, the protocol is both cost-effective and scalable. It omits expensive agar, sucrose, and sterile room maintenance; reduces labor (no agar washing or individual potting); and shortens the production cycle by at least one month. Similar cost and time savings have been reported for other medicinal plants using ex vitro systems [26].

Most importantly, this protocol addresses the urgent conservation needs of *M. savatieri*, whose natural populations have declined sharply due to habitat destruction and over-harvesting [5]. It enables rapid, host-free production of high-quality plantlets for reintroduction, ex situ conservation, and artificial cultivation. The integrated approach—moderate auxin pulse plus balanced organic substrate—is cost-effective, time-efficient and readily scalable, offering a practical solution for both conservation and commercial production. Moreover, the quadratic regression framework developed here may provide a useful basis for similar optimization efforts in other endangered root hemiparasitic plants, and future work should test its applicability to additional Scrophulariaceae species with comparable rooting difficulties.

Several methodological limitations should be acknowledged. The PGR, immersion time and substrate optimizations were performed sequentially, so potential interactions among these factors were not evaluated. The protocol was validated only under greenhouse conditions and over a single season, and the physiological mechanisms discussed are inferred from previous reports rather than directly measured. Addressing these limitations, future work should investigate (1) the combined effects of PGRs and substrates using a factorial design, (2) field performance and long-term establishment, and (3) the underlying hormonal and molecular changes during rooting.

## 4. Materials and Methods

### 4.1. Materials

All *M. savatieri* material originated from Dongyuan village, Fenyi County (coded as FY), located in Jiangxi Province, China [5]. Living plants were immediately transferred to the experimental center of subtropical forestry, Chinese Academy of Forestry. Shoots of uniform height (~2 cm) with a minimum of four leaves obtained from wild seedlings were used as explants. The method of surface sterilization used was described by Li et al. [7]. In vitro shoots (~2 cm) with two internodes were inoculated on modified MS media containing BA (0–0.5 mg·L^−1^) and GA_3_ (0.2–0.4 mg·L^−1^) [7]. This proliferation stage (45 days, 25 ± 2 °C, 16 h photoperiod) served to generate uniform microshoots and improve shoot vigor before root induction; it replaces neither the subsequent ex vitro rooting step nor contributes to the claimed time savings, which arise solely from eliminating the in vitro rooting phase (agar, sucrose, activated charcoal). After 45 days of culture, shoots were transferred to cultivation medium.

### 4.2. Treatment, Measurement and Analysis

To investigate early tissue culture seedling survival rate and growth of *M. savatieri*, one in vitro trial and two ex vitro trials were carried out under greenhouse conditions, located in the Experimental Center of Subtropical Forestry, Chinese Academy of Forestry (CAF) in the city of Fenyi, Jiangxi, with an altitude of 78 m and coordinates 27°49′ N and 114°39′ E. A mean luminosity of 224 µmol·m^−2^·s^−1^ was recorded, with mean maximum and minimum temperatures of 34 °C and 26 °C, respectively, and relative humidity ranging from 55% to 80%.

#### 4.2.1. In Vitro Rooting Procedure and Acclimatization

Single shoots of FY, taken from shoot clumps cultured in vitro on modified MS medium containing BA (0–0.5 mg·L^−1^) and GA (0.2–0.4 mg·L^−1^) for 1 month were used as explants in this experiment. Six shoots were placed in a glass bottle (150 mL) with a vented lid containing charcoal supplemented rooting medium (DCR basal salt mixture, 2% edible sugar, 1.0 g·L^−1^ activated charcoal (separately autoclaved in a glass bottle), 5.8 g·L^−1^ agar and brought to pH 5.8 with 1 N KOH), containing 0.2–1.0 mg·L^−1^ ABT, NAA or IAA. Activated charcoal was added after the medium cooled to 60 °C or below, preventing it from settling to the bottom and leaving the medium clear.

Agar was used as the supporting material during the in vitro rooting experiment. The rooting medium supplemented with 1.0 mg·L^−1^ ABT was added to the agar medium before autoclaving, in order to provide the auxin for rooting. After 25 days of culture, shoots were taken from the glass bottles, and the roots were rinsed with water to eliminate the culture medium. Thirty shoots from each treatment were investigated, including rooting frequency (the percentage of microshoots that produced visible adventitious roots in vitro after 25 days of culture), root number, and root length, as well as plant height (Table 1). In preliminary experiments, shoots cultured on rooting medium without PGR (0 mg·L^−1^) produced predominantly aerial roots and failed to develop transplantable root systems. Therefore, the 0-control condition was not included in the formal factorial design, as the primary aim of this stage was to screen PGR types and concentrations that yield functional roots for subsequent ex vitro experiments.

#### 4.2.2. Ex Vitro Rooting and Acclimatization

To overcome the problem of aerial root development in vitro, ex vitro rooting was tested using two separate methods. Based on the in vitro screening results (Section 2.1; Table 1), 1.0 mg·L^−1^ ABT was identified as the optimal auxin treatment, but the optimal NAA/IAA concentrations and immersion duration for ex vitro application remained to be determined. To ensure terminological clarity throughout this study, the following definitions apply: (1) “Survival rate” refers to the percentage of transplanted plantlets that remained green and showed active shoot growth at a given time point (recorded at 45 days after transplantation). (2) “Seedling rate” refers to the percentage of the original transplanted plantlets that successfully overwintered and resumed normal growth in the following spring; this term does not refer to seed-derived progeny.

Experiment 1: Optimization of PGR concentration and immersion duration.

*M. savatieri* clone *ZJLS11* was selected as the starting material for ex vitro research, using micro-shoots obtained from a stabilized culture started from stem in vitro [5]. This clone was chosen for PGR screening due to its vigorous growth and sufficient microshoot supply. Such shoots showed good performance at the stage of proliferation but lacked a root, and they were passaged at least 1 cycle on PGR-free media. The micro-shoots (5–6 cm) were individually excised and dipped in freshly prepared auxins solutions (300, 600, 900, 1200, 1500, 1800 mg·L^−1^ NAA and IAA, a ratio of 2 to 3) and GA_3_ (Shanghai Yuanye Bio-Technology Co., Ltd., Shanghai, China, ≥90% purity) (50, 100, 150, 200, 250, 300 mg·L^−1^) for various durations (15 s, 30 s and 60 s), in which the auxins were chosen based on the results of the in vitro rooting experiment. The full factorial combinations of these concentrations and immersion times are presented in Table 2 (Results). The auxin-treated shoots were transplanted into non-woven fabric bags (50 mm diameter × 80 mm height) containing potassium permanganate solution (*w*/*v* 0.1%) sterilized substrates [a mix of peat moss, sawdust, carbonized chaff and expanded perlite (3:3:3:1)], with 1 plant per container. The bags were covered with plastic film to maintain temperature and humidity for ex vitro acclimatization.

In this experiment, a two-factorial (concentration of PGR and soaking time) completely randomized block design was conducted with three replications, each block contained 66 plants, thus a total of 3564 plants (66 plants × 18 treatments × 3 replications) for each clone. 

The experiment lasted for 7 months. The studied features included survival rate, length of the longest stems, number and length of the roots (mm), area and volume of the roots, and the fresh weight of the plants (g).

Experiment 2: Optimization of substrate composition.

After optimizing for the best PGR combination (treatment 10: NAA 480 + IAA 720 + GA_3_ 200 mg·L^−1^, 15 s immersion), the effect of substrates on ex vitro rooting was evaluated. For rooting under ex vitro conditions, 12 substrate formulations (compositions listed in the footnote of Table 3) were evaluated in this experiment and 198 shoots were planted into each substrate (2376 shoots total, 198 bags for each substrate). The shoots (5–6 cm) of clone FY were individually derived from multiplied cultures as in the previous study. Clone FY (the local ecotype targeted for conservation) was used for substrate optimization, as this experiment focused on physical substrate properties rather than genotypic responses to PGRs; no genotype-dependent differences were expected for substrate physical responses. The shoot base (0.5–1.0 cm) was first soaked in the fungicide solution at a concentration of 1 g·L^−1^ for 5 min, after which the shoots were placed on trays to drain. After 1–2 h, the shoot base was dipped again in freshly prepared solutions supplemented with auxins, using the optimized combination identified before.

The substrate physical properties are presented in Table 3 which were measured as previously shown by Pu et al. [45] and *EN 13041:1999* [46] with slight modifications. Peat moss substrate was supplied in 20 L bags, whereas other substrates were supplied in 3 L bags. Water retention was determined at saturation and after submitting the samples for a 48 h equilibrium period. A plastic beaker with known volume (V ≥ 4 L, mark the 3 L line and chisel a small gap with knife) was taken, and its net weight (W1) was determined; the plastic beaker was filled to the 3 L line with the naturally dried substrate and weighed again (W2). Then, the plastic beaker containing the substrate was sealed with two layers of wet gauze, and the chiseled gap was sealed with waterproof tape. After submerging for 48 h in water, the plastic beaker was taken out and the sealing tape was removed, letting the water above the 3 L line overflow freely; the water-saturated weight was determined (W3), as well as the weight of the wet gauze for sealing (W4). Finally, the plastic beaker was wrapped, letting the water in the beaker (gravity water) drain freely and the final weight was taken (W5). The physical index was then calculated according to the following formula:

Bulk density (*BD*) = (W_2_-W_1_)/3000; Water holding capacity (%) (*θ_f_*) = (W_5_ − W_1_ − W_4_)/(W_2_ − W_1_) × 100; Total porosity (%) (*TP*) = (W_3_ − W_2_)/3000 × 100; Aeration porosity (%) (*AFP*) = (W_3_ + W_4_ − W_5_)/3000 × 100; Porosity and water-holding (%) (*WFP*) = *TP* − *AFP*. 

The experiment was carried out as a randomized complete block design with three blocks under 12 different substrates.

Plantlets in two trials were grown in a semi-controlled light and temperature greenhouse. During the experimental period, the average daily light intensity, temperature and relative humidity of the greenhouse ranged from 72 to 376 µmol photon m ^−2^ s ^−1^, 26 to 34 °C and 55 to 80%, respectively. The presence of fungi was controlled with Carbendazim sprayings and, after 20 days, the plastic mulch was opened gradually over a week-long period. Afterward, according to the maximum water-holding capacity of the substrate we measured, we used 50% of the capacity as the daily irrigation standard. Soil water content was then maintained during the experiment by watering trays to the 50% capacity with water every other day. Tray positions were moved randomly by hand within each block every 2 weeks throughout the experiment to remove any edge effects. The survival rate was recorded 45 days after transplanting, and plants were harvested six months later for root trait measurements (root length, area, volume, and diameter). The long-term seedling (establishment) rate was recorded in the following spring after overwintering. In addition, the number of new buds per surviving plantlet was counted during the same period. Furthermore, five plants were randomly selected from each block. These whole plants were immersed in tap water and washed carefully by hand to remove substrate mixture from fine roots. Subsequently, root tip number, root length, area and volume were measured immediately after harvesting using an Epson Perfection V800 scanner and the WinRHIZO scanner program. In this system, the ‘root tip number’ refers to the total count of all terminal meristems of both primary and lateral roots detected by the scanner software, not the number of adventitious roots arising from the stem base.

### 4.3. Statistical Analysis

Data were analyzed by one-way analysis of variance (ANOVA) using SPSS 19.0 (SPSS Inc., Chicago, IL, USA). For experiments involving two categorical factors (auxin type × concentration for in vitro rooting; PGR concentration × immersion time for ex vitro rooting), two-way analysis of variance (two-way ANOVA) was performed to evaluate main effects and interactions. All factorial analyses were conducted using the General Linear Model (GLM) procedure in SPSS. Significant differences among treatment means were determined by Duncan’s multiple range test at *p* < 0.05. Percentage data were arcsin-square-root transformed prior to analysis to satisfy normality assumptions.

To examine the relationships between root traits (length, area and diameter) and ex vitro survival rate, quadratic polynomial regression models were fitted using the ordinary least-squares method. Model significance was assessed by F-test, and goodness-of-fit was evaluated by the coefficient of determination (*R*^2^). All regression analyses were performed using the Regression module in SPSS.

## 5. Conclusions

This study has successfully developed a complete and efficient ex vitro rooting and acclimatization system for the endangered medicinal herb *M. savatieri*. We systematically optimized the key factors affecting post-transplant survival: the optimal plant growth regulator treatment was a 15 s immersion in a solution containing NAA 480 mg·L^−1^, IAA 720 mg·L^−1^ and GA 200 mg·L^−1^ (treatment 10), resulting in a four-month survival rate of 95.45% and a next-year seedling rate of 92.96%. The most suitable substrate was T12 (peat moss:perlite:sawdust:carbonized chaff = 3:1:3:3, by volume), which achieved a survival rate of 85.20%. Quadratic regression analysis revealed that root length is the best predictor of survival, with an optimum of 94.7 mm. This integrated protocol eliminates the problematic in vitro rooting phase, avoids aerial root formation, reduces cost and labor, and shortens the production cycle by at least one month. Under the greenhouse conditions tested, it may provide a practical and scalable basis for the propagation of *M. savatieri*, and could potentially support ex situ conservation and artificial cultivation efforts for this threatened species, although its performance under field conditions and long-term establishment remain to be evaluated.

## 6. Patents

Li, F.Q.; Zeng, M.S.; Liu, S.Z.; Zhu, X.M.; Luo, G.S.; Zou, Y.L. Method for rooting tissue culture seedlings of medicinal plant *Monochasma savatieri* outside bottle. Chinese Patent Application No. CN202211580480.4, 2022. (pending).

## Figures and Tables

**Table 1:** Effect of ABT, IAA and NAA concentrations on in vitro rooting of *M. savatieri*.

Concentrations of PGRs		Rooting Frequency (%)	Number of Roots/Explant	Root Length (cm)	Plant Height (cm)
Auxin	Concentration (mg·L^−1^)				
ABT	0.2	32.97 ± 4.52 ab	1.26 ± 0.06 c	2.07 ± 1.77 b	5.43 ± 2.61 abc
	0.4	38.12 ± 3.40 ab	2.03 ± 0.34 ab	1.21 ± 0.84 b	3.61 ± 0.28 c
	0.8	41.50 ± 12.58 ab	1.43 ± 0.16 bc	0.76 ± 1.36 b	3.94 ± 1.18 bc
	1	61.66 ± 20.54 a	2.11 ± 0.56 a	1.68 ± 1.13 b	4.41 ± 1.22 abc
IAA	0.2	37.99 ± 9.25 ab	1.87 ± 0.71 abc	1.06 ± 1.19 b	4.97 ± 0.86 abc
	0.4	40.43 ± 13.23 ab	1.75 ± 0.39 abc	1.11 ± 0.89 b	4.61 ± 0.65 abc
	0.8	42.48 ± 15.16 ab	1.72 ± 0.41 abc	1.14 ± 1.11 b	4.97 ± 0.81 abc
	1	38.24 ± 13.20 ab	2.05 ± 0.26 ab	5.21 ± 2.95 a	6.09 ± 2.07 a
NAA	0.2	30.51 ± 11.91 b	1.66 ± 0.21 abc	0.38 ± 0.40 b	5.67 ± 0.44 ab
	0.4	35.75 ± 8.45 ab	1.76 ± 0.26 abc	0.56 ± 0.45 b	5.12 ± 0.36 abc
	0.8	38.81 ± 16.31 ab	1.66 ± 0.33 abc	0.29 ± 0.12 b	5.55 ± 0.50 ab
	1	42.11 ± 10.77 ab	1.56 ± 0.10 abc	0.45 ± 0.14 b	6.01 ± 0.67 a

Different letters within the same column indicate significant differences at *p* < 0.05 by Duncan’s multiple range test.

**Table 2:** Influence of NAA, IAA and GA on ex vitro rooting of plantlets of *M. savatieri* (clone *ZJLS11*).

Treatments	NAA (mg·L^−1^)	IAA (mg·L^−1^)	GA (mg·L^−1^)	Immersion Time (s)	Number of Root Tips	Root Length (mm)	Root Area (cm^2^)	Root Volume (cm^3^)	Root Diameter (cm)
1	120	180	50	15	95.50 ± 20.23 c	71.80 ± 23.11 de	7.62 ± 2.13 bc	0.08 ± 0.02 cd	0.32 ± 0.03 bc
2	120	180	50	30	116.67 ± 50.27 bc	55.30 ± 18.80 ef	4.79 ± 1.76 cd	0.04 ± 0.02 de	0.26 ± 0.01 ef
3	120	180	50	60	307.50 ± 157.72 a	49.61 ± 10.90 ef	4.07 ± 0.74 d	0.03 ± 0.01 e	0.26 ± 0.02 ef
4	240	360	100	15	120.50 ± 52.82 bc	70.44 ± 30.44 de	7.25 ± 3.31 bc	0.07 ± 0.04 cd	0.30 ± 0.04 cd
5	240	360	100	30	149.38 ± 95.17 bc	79.06 ± 44.39 cd	7.27 ± 3.60 bc	0.07 ± 0.03 cd	0.28 ± 0.03 ef
6	240	360	100	60	132.33 ± 48.24 bc	69.57 ± 27.25 de	7.39 ± 2.75 bc	0.08 ± 0.03 cd	0.33 ± 0.02 bc
7	360	540	150	15	117.17 ± 47.03 bc	95.84 ± 37.57 b	9.15 ± 4.36 ab	0.09 ± 0.05 b	0.32 ± 0.03 bc
8	360	540	150	30	163.17 ± 54.49 bc	50.48 ± 11.52 ef	4.57 ± 1.15 cd	0.04 ± 0.02 de	0.29 ± 0.06 ef
9	360	540	150	60	105.00 ± 28.47 bc	65.86 ± 22.35 de	5.92 ± 2.17 bc	0.05 ± 0.02 cd	0.30 ± 0.02 de
10	480	720	200	15	101.17 ± 33.97 bc	125.49 ± 50.14 a	9.20 ± 3.76 ab	0.07 ± 0.04 cd	0.23 ± 0.03 f
11	480	720	200	30	101.00 ± 19.78 bc	76.84 ± 20.46 cd	8.17 ± 2.06 ab	0.09 ± 0.03 bc	0.34 ± 0.03 bc
12	480	720	200	60	124.83 ± 41.05 bc	87.13 ± 28.87 bc	11.48 ± 4.41 a	0.17 ± 0.08 a	0.41 ± 0.06 a
13	600	900	250	15	137.00 ± 48.92 bc	55.47 ± 22.31 ef	4.23 ± 1.44 d	0.03 ± 0.01 e	0.24 ± 0.06 ef
14	600	900	250	30	151.17 ± 48.69 bc	45.58 ± 13.44 fg	5.12 ± 1.47 cd	0.05 ± 0.02 cd	0.35 ± 0.03 b
15	600	900	250	60	189.83 ± 48.04 b	40.54 ± 10.35 g	4.64 ± 1.47 cd	0.05 ± 0.02 cd	0.35 ± 0.06 b
16	720	1080	300	15	123.33 ± 51.89 bc	55.86 ± 31.02 ef	6.09 ± 3.31 bc	0.07 ± 0.04 cd	0.34 ± 0.04 b
17	720	1080	300	30	110.33 ± 61.01 bc	51.91 ± 29.00 ef	5.15 ± 3.65 cd	0.05 ± 0.05 cd	0.29 ± 0.07 de
18	720	1080	300	60	117.50 ± 59.95 bc	60.78 ± 11.83 de	5.84 ± 1.78 bc	0.06 ± 0.03 cd	0.28 ± 0.05 ef

Data are means ± SD. Different letters within the same column indicate significant differences at *p* < 0.05 by Duncan’s multiple range test.

**Table 3:** Physical properties of the twelve substrate formulations. Values are means of three replicates.

Substrates	Bulk Density (g/cm^3^)	Water-Holding Capacity (*v*/*v*)	Total Porosity (g/g)	Aeration Porosity (%)	Water-Holding Porosity (%)	Void Ratio
T1	1.10	179.69	105.50	18.24	87.26	0.209
T2	0.95	202.55	113.50	16.08	97.42	0.165
T3	0.56	310.28	140.50	22.75	117.76	0.193
T4	0.51	369.30	145.00	7.66	137.34	0.056
T5	0.47	329.47	140.00	32.15	107.85	0.298
T6	0.43	360.54	149.50	38.77	110.73	0.350
T7	0.32	563.43	154.00	5.70	148.30	0.038
T8	0.58	326.53	136.00	5.74	130.26	0.044
T9	0.57	324.33	137.50	10.75	126.75	0.085
T10	0.62	305.13	136.00	8.82	127.18	0.069
T11	0.31	442.48	149.00	44.54	104.46	0.426
T12	0.45	420.36	160.00	17.44	142.56	0.122

T1–T12 substrate formulations (volume ratios, *v*/*v*) are as follows: T1 = 10 peat moss; T2 = 5 peat moss:3 sawdust:2 carbonized chaff:5 popcorn soil; T3 = 6 peat moss:3 sawdust:2 carbonized chaff:1 soil; T4 = 3 peat moss:3 sawdust:2 carbonized chaff:1 popcorn soil:1 pine needle; T5 = 3 peat moss:3 sawdust:2 carbonized chaff:1 soil:1 bark; T6 = 3 peat moss:1 perlite:4 sawdust:2 carbonized chaff; T7 = 3 peat moss:1 perlite:3 sawdust:2 carbonized chaff:1 bark; T8 = 4 peat moss:3 sawdust:2 carbonized chaff:1 popcorn soil; T9 = 4 peat moss:3 sawdust:2 carbonized chaff:1 sand; T10 = 2 peat moss:3 sawdust:2 carbonized chaff:1 popcorn soil:1 bark:1 pine needle; T11 = 3 peat moss:1 perlite:3 sawdust:3 carbonized chaff; T12 = 3 peat moss:1 perlite:3 sawdust:3 carbonized chaff.

**Table 4:** Quadratic regression parameters (forced through origin) for root traits on survival rate of *M. savatieri*.

Independent Variable	*R* ^2^	*F* Value	*p* Value	Optimum Value	Maximum Predicted Survival Rate (%)
Root length	0.977	344.64	<0.001	94.7 mm	98.6
Root area	0.976	334.43	<0.001	8.15 cm^2^	92.4
Root diameter	0.984	497.09	<0.001	0.35 cm	86.7

## Data Availability

The data presented in this study are available on request from the corresponding authors. The data are not publicly available due to privacy and ethical restrictions.

## Supplementary Materials

The following supporting information can be downloaded at: https://www.mdpi.com/article/10.3390/plants15172638/s1, Table S1: Two-way ANOVA (*F* values) for the effects of auxin type, concentration, and their interaction on in vitro rooting parameters of *M. savatieri.*; Table S2: Two-way ANOVA (*F* values) for the effects of PGR concentration, immersion time, and their interaction on ex vitro rooting performance of *M. savatieri* (*ZJLS11*).
