# Supplementary material for: Establishment of an Ex Vitro Rooting Strategy for Monochasma savatieri Increases Propagation Efficiency
Source: Plants (Basel). 2026 Aug 28;15(17):2638. doi: 10.3390/plants15172638 (PMC13567222; doi:10.3390/plants15172638)
Supplement: Supplementary file 1 [file plants-15-02638-s001.zip › plants-4434195-supplementary.pdf]

Table S1. Two-way ANOVA (*F* values) for the effects of auxin type, concentration, and their interaction on in vitro rooting parameters of *M. savatieri*.

Source of Variation	Rooting Frequency	Number of Roots	Root Length	Plant Height
Auxin type	1.12	11.36 **	9.26 **	6.12 *
Concentration	0.10	2.17	4.90 *	2.73
Auxin type × Concentration	2.84*	1.00	3.92 *	0.74

* $p < 0.05$; ** $p < 0.01$ (Duncan's multiple range test). Given the suboptimal performance of in vitro rooting, we next explored an ex vitro rooting strategy.

Table S2. Two-way ANOVA (*F* values) for the effects of PGR concentration, immersion time, and their interaction on ex vitro rooting performance of *M. savatieri* (*ZJLS11*).

Source of Variation	Survival Rate	Seedling Rate	Bud Number	Plant Height
Concentration	16.83 **	15.46 **	3.43 *	6.24 **
Immersion time	2.36	3.04	1.91	4.53 *
Concentration × Immersion time	9.81 **	12.48 **	1.86	4.85 **

* $p < 0.05$; ** $p < 0.01$ (Duncan's multiple range test).
